# Innovation and efficiency in financial institutions

**DOI:** 10.3389/frma.2022.805116

**Published:** 2022-08-10

**Authors:** Vania Sena, Amangeldi Kenjegaliev, Aliya Kenjegalieva

**Affiliations:** ^1^Management School, The University of Sheffield, Sheffield, United Kingdom; ^2^Hull University Business School, University of Hull, Kingston upon Hull, United Kingdom; ^3^Department of Economics, Faculty of Humanities and Social Sciences, University of Bath, Bath, United Kingdom

**Keywords:** efficiency, distance function, innovation, ranks, MCDA

## Abstract

This paper proposes a new methodology that combines standard production theory with Multiple-Criteria Decision Analysis (MCDA) methods to rank banks based on their capability of using investment in new technologies to reduce the other inputs' usage, for a given level of output. Banks are first ranked based on their investment in innovation (innovation rank); afterwards, we calculate the overall rank by combining two factors of production, viz. labor and assets, using the PROMETHEE II approach that belongs to the family of the outranking methods. We then use directional efficiency measures to measure the banks' efficiency by means of relation between two ranks, for a given level of the outputs. We apply the methodology to a sample of US and EU banks sourced from Orbis BankFocus. The key findings suggest there are four types of banks in our sample: (a) banks whose innovation rank is positively correlated with the overall rank; (b) banks exhibiting a negative correlation between two ranks: their overall ranks are low while still exhibiting high innovation ranks; (c) banks with high overall rank but low innovation rank and (d) banks with the worst ranks both for the innovation rank and the overall rank. The least efficient banks belong to this group.

## Introduction

Over the last twenty years, the banking sector has invested in digital technologies (Rishi and Saxena, 2004) that have changed their production processes and the way they interact with customers. Digital technologies are a type of innovation that allow banks to process and transmit information easily. As a result, banks have been able to expand the range of products they offer as well as the menu of ancillary services associated to them. However, the impact of investment in digital technologies is not only limited to the outputs of the banks. Like any other type of innovation, digital technologies reduce the demand for other types of inputs such as labor and capital: indeed, tasks which were routinely performed by employees of a branch can now be performed by bots or other information processing technologies. As the demand for labor reduces, there is less need for a bank to invest in physical buildings and as a result, the demand for fixed assets decreases as well.

The impact of the investment in digital technologies on the demand of the other inputs creates some theoretical difficulties when trying to model banks' production process and measuring their efficiency. Indeed, digital technologies (like any other types of innovation) tend to be considered inputs of the banks' production function. However, investment in digital technologies does not behave like any other inputs with the result that assumptions such as strong disposability of inputs may not be realistic when modeling production function where investment in digital technologies is one of the inputs. Indeed, on the one hand, the increase of the expenditure on digital technologies may lead to an increase of the outputs for a given level of inputs' usage. On the other hand, the same increase of the innovation expenditure may lead to a decrease of the inputs' usage for a given level of output. As a result, it is unclear whether we can assume that banks can still produce the same amount of outputs if all the inputs shrink simultaneously. This conceptual weakness creates problems when using standard methodologies for the measurement of efficiency. For instance, Data Envelopment Analysis (DEA) relies on strong disposability of inputs and any attempt to remove this assumption (for instance, by introducing the concepts of weak disposability) has led to other issues (see Podinovski et al., 2009).

To circumvent this problem when measuring the efficiency of banks while allowing for the investment in digital technologies as an input of the production function, we propose a new methodology that combines standard production theory with MCDA methods to rank banks based on their capability of using investment in digital technologies to reduce the other inputs' usage, for a given level of output. We start by considering a directional distance function which is a standard representation of a technology and is related to both the input distance function (which assumes that outputs are exogenous) and the output distance function (which assumes the exogeneity of the inputs). Assuming that outputs are exogenous, banks have two types of inputs: (a) labor and capital, that can be decreased for a given level of outputs in the case of inefficient banks and (b) expenditure in digital technologies that may have to stay constant (or increase) for a given level of output even in the case of inefficient banks. To capture the fact that contractions of inputs may be associated to increases of the remaining input, we propose to replace the inputs with ranks which capture the fact that the inputs move in different directions.

To do so, the investment in innovation is separated from the other inputs. Banks are first ranked according to the size of investment in innovation (innovation rank); afterwards, we calculate the overall rank by combining two factors of production, viz. labor and assets, using the PROMETHEE II approach (Brans et al., 1986) that belongs to the family of the outranking methods. We then use directional efficiency measures to measure the banks' efficiency by means of relation between two ranks, for a given level of the outputs. We would expect that improvements along the overall rank (i.e., reduction in the inputs' usage) may be accompanied by proportional improvements along the innovation rank. However, if this is not the case, then the innovation ranks are not identical for banks with equivalent overall ranks implying that increases in the investment in digital technologies does not translate in an equivalent reduction of the inputs' usage for a given level of outputs.

To exemplify our approach, we consider three models. The first one focuses on investment on intangible assets as a proxy of innovation (proxied by intangible assets). The second one focuses on non-performing loans (a ratio of impaired loans to gross customer loans and advances) and allows to illustrate how our approach can be used for other types of variables. Finally, the third model focuses on a sample drawn from a Gaussian distribution. We have chosen the latter two as a robustness check of our approach. For example, non-performing loans have significant impact on bank performance and asset quality (see for example Simoens and Vennet, 2021). Deteriorations in non-performing loans reduces bank competitiveness and performance. Hence, we expect the least productive banks to have the lowest ranks both for non-performing loans and the overall performance (and vice versa in the case of the most efficient bank). Our approach builds on Ishizaka et al. (2018) although to the best of our knowledge, combining MCDA methods with standard production theory to measure efficiency in the banking industry has never been done.

The remainder of the paper is organized as follows. In the next section, we discuss production technology set and provide details of the directional distance function. In section on production technology and ranks, we introduce our ranked distance function. First, we will explain how to obtain our ranks: the innovation rank and the overall rank. Then we focus on the ranked distance function that we use in this paper. Section Empirical analysis: data, operationalization and methodology is devoted to data and data analysis. Section Estimation results focuses on our results and the robustness checks. Finally, the last section “Conclusions” offers final remarks.

## Measuring efficiency using frontier analysis

As standard in the operational research literature, we begin from defining the technology available to the DMUs. The technology is characterized by an attainable set *T* (i.e., the production frontier) that includes all combinations of inputs and outputs that are technically achievable. Now, let an entity *b* with *b* = 1, .., *B* has a vector *x*_*b*_ with *K* inputs, indexed *k* = 1, …, *K*, at its disposal. Let *y*_*b*_ be a vector of *U* outputs, indexed *u* = 1, …, *U* that the entity *b* produces.

The production technology, in that case, is characterized by:


(1) $$\begin{array}{l} T=\left\{\left(x_{b},y_{b}\right)\in {\mathbb{R}}^{K+U}:x_{b}\;can\;produce\;y_{b}\right\} \end{array}$$

where *T* describes a set of input vectors that are feasible for each output vector (Glass et al., 2020). It is usually assumed that (a) *T* is convex, (b) *T* is closed, and (c) there is free disposability of inputs and outputs (see Chambers et al., 1998; Daraio and Simar, 2005; Atkinson and Tsionas, 2016; Kuosmanen and Johnson, 2017; and Layer et al., 2020).

One of the most flexible approaches in productivity literature is a directional distance function introduced by Chambers et al. (1998) and based on Luenberger's (1992) benefit function. Given a directional vector of inputs and outputs, the directional distance is defined as:


(2) $$\begin{array}{l} \beta \left(x_{b},y_{b}:d_{x},\;d_{y}\right)=sup\left\{\beta >0|\left(x_{b}-\beta d_{x},\;y_{b}+\beta d_{y}\right)\in T\right\} \end{array}$$

In this case, the distance from the efficient frontier is estimated in an additive way and the direction to the frontier is defined by *d*_*x*_ and *d*_*y*_. This approach is popular among researchers because it explicitly allows to set some elements in the directional vector equal to zero (see for example Fare and Grosskopf, 2010). In a recent paper, Daraio and Simar (2014) point out that the efficient frontier is uniquely defined by the boundary of the attainable set *T* (where all the inputs and outputs are involved), but the distance to the frontier depends on the chosen direction. The direction can be different for each entity (like in the radial cases) or it can be the same for all the decision-making units. According to Daraio and Simar (2014), this way of measuring the distance is very flexible and generalizes the “oriented” radial measures proposed by Farrell (1957).

There is a large empirical literature on how to measure efficiency of organizations using frontier analysis (see for example, Chambers et al., 1998; Fare and Grosskopf, 2010; Duygun et al., 2013, 2016; Glass et al., 2014; Glass and Glass, 2018). Frontiers can be computed using either econometric techniques or Data Envelopment Analysis (DEA) (Banker et al., 1984). Econometric techniques assume that the most productive firms are located on the production curve. However, often these estimations are imprecise and prone to errors (Land et al., 1993; Fernandez et al., 2000; Layer et al., 2020). On the contrary, DEA is a very intuitive technique that measures DMU's efficiency as the ratio between the sum of the weighted output levels and the sum of the weighted input levels. The advantages of DEA over econometric techniques are multiple: first, it does not require any assumption on the functional form of the distance frontier. Second, it can accommodate multiple inputs and outputs. However, several authors have pointed out that one limitation of DEA is that it assumes strong (free) disposability of inputs and outputs. The assumption of free disposability of outputs means that for any levels of inputs, it is possible to reduce the level of outputs freely; conversely, free disposability of inputs implies that for a given level of outputs, inputs can be increased freely. The assumption of strong disposability is incorporated in the constant (CRS) and variable (VRS) returns-to-scale DEA models (Banker et al., 1984).

The assumption of strong disposability of inputs and/or outputs is unrealistic in some contexts. A lot of literature has focused on the case of undesirable outputs whose reduction has to be accompanied by a decrease of desirable outputs. However, this is also true in the case of inputs: for instance, an expansion of some inputs may be costly if DMUs are required to invest in some other inputs simultaneously. Traditionally, these cases are accommodated by introducing the assumption of weak disposability of inputs (Shephard, 1970). The assumption of weak *input* disposability states that, if a vector of outputs is produced from a vector of inputs, then the same vector of outputs can be produced if we scale up all components of the input vector in the same proportion. This assumption is problematic on two counts: first, at a theoretical level. Kuosmanen and Podinovski (2009) show that in these cases the production technology is a convex set. Second, the assumption may not be useful for DMUs which have to simultaneously increase some inputs while decreasing others, to be able to produce the same level of outputs. For instance, when modeling the production technology of commercial banks, assuming that labor and capital may decrease freely for a given level of outputs may be problematic as such decreases are usually the result of increases of the investments in digital technologies—inputs of the commercial banks' production technology. In these cases, alternative methodologies may be needed to model these technologies.

## Production technology and ranks

Our approach starts from the observation that there are industries where DMUs have to minimize some inputs (say, I_min) while maximizing others (I_max) for a given level of outputs; if so, we can identify the optimal combination of I_min and I_max, for each DMU and for given outputs. As in the case of the frontier analysis, we can calculate the optimal combination of I_min and I_max for the whole industry and this will give us a benchmark against which assess the performance of each DMU in the industry. Our approach starts by splitting inputs into two groups i.e., inputs that need to be reduced and inputs that need to be expanded. As we deal with multiple inputs in each case, we propose to convert them into ranks which will be used instead of the inputs. The use of ranks strips measurement units from the variables, reduces the dimension of the dataset and avoids the need of additional assumptions to measure efficiency.

In the case of one variable, the rank is defined as


(3) $$\begin{array}{l} r_{b1}=rank\left({b:x}_{b1}\right) \end{array}$$

Since the rank in (3), *r*_*b*1_, is based on a single variable, the DMU with the largest value is allocated the rank *r*_*b*1_ = 1. However, if we have inputs with different measurement units and scales, we need to use alternative methodologies to calculate the ranks. In these cases, MCDA methods which allow to rank multiple alternatives based on a number of decision criteria are particularly useful as they allow to collapse multi-dimensional datasets into a single index.

One of them is an outranking method that uses a pairwise comparison of alternatives *via* a preference index so that an alternative *x* is claimed to outrank another alternative *y* if, and only if, *x* is at least as good as *y* and there is no strong argument to contradict this assertion. For our analysis, we use the PROMETHEE II approach (Brans et al., 1986). Assume that a set of criteria be *z*_*b*_ = (*x*_*b*2_, …, *x*_*bK*_) where *x*_*b*2_, …, *x*_*bK*_ are *K* − 1 inputs. The comparison among alternative combinations of inputs is in terms of preferences. According to Brans et al. (1986), the preference function for each element *i* in *z*_*b*_ is given by *P*_*i*_(*b, c*), where *b* ≠ *c*. This function represents the preference intensity of the DMU *b* over the DMU *c* with respect to element *i*. For each criterion the preference function is a non-decreasing function of


(4) $$\begin{array}{l} P_{i}\left(b,c\right)=\Phi \left\{b\left(i\right)-c\left(i\right)\right\} \end{array}$$

Brans et al. (1986) report six types of generalized criteria evaluations. We use a modified usual criterion since it gives us binary outcome and we only need to decide between two types: either *P*_*i*_(*b, c*) = 0 if Φ{*b*(*i*)−*c*(*i*)} ≤ 0 or *P*_*i*_(*b, c*) = 1 if Φ{*b*(*i*)−*c*(*i*)} > 0 (i.e., strict preference of *b* over *c*).

Each element *i* is given a weight, *w*(*i*), which indicates the relative importance of it for a decision maker. In the absence of a preferred choice the criterion weight is *w*(*i*) = (*K* − 1)^−1^. Then the preference index, 𝔓, is given as


(5) $$\begin{array}{l} 𝔓\left(b,c\right)=\underset{i\in \left(K+U-1\right)}{\sum }w\left(i\right)P_{i}\left(b,c\right) \end{array}$$

As Brans et al. (1986) note, 𝔓(*b, c*) is a combined intensity preference for the entity *b* over the entity *c* taking into account all element *i* in *z*_*b*_. The resulting outcome takes the value between 0 [weak preference of the entity *b* over the entity *c* if 𝔓(*b, c*) close to 0] and 1 [strong preference of the entity *b* over *c* if 𝔓(*b, c*) close to 1].

For each entity *b* the outranking character (outflow) is given by


(6) $$\begin{array}{l} \phi^{+}\left(b\right)=\underset{c\in B}{\sum }𝔓\left(b,c\right) \end{array}$$

The outflow computed in (6) indicates preference of *c* ∈ *B* compared to *b*. Equivalently for outranked character (entering flow) of *b* is:


(7) $$\begin{array}{l} \phi^{-}\left(b\right)=\underset{c\in B}{\sum }𝔓\left(c,b\right) \end{array}$$

The inflow computed in (14) indicates preference of *b* compared to *c* ∈ *B* with the net flow being equal to:


(8) $$\begin{array}{l} \phi \left(b\right)=\phi^{+}\left(b\right)-\phi^{-}\left(b\right) \end{array}$$

For each pair of DMUs, we have two possibilities:


(9) $$\begin{array}{l} \{\begin{array}{cc} b\;outranks\;c, & iff\;\varphi \text{(}b\text{)}>\phi (c)\\ b\;is\;indifferent\;to\;c, & iff\;\varphi \text{(}b\text{)}=\phi (c) \end{array} \end{array}$$

This partial ranking approach transforms separate distributions of each element in $\left(x_{bk}-x_{b1}\right)\in {\mathbb{R}}^{K-1}$ into a single composite variable with a uniform distribution regardless of the asymmetries in the original distributions. Therefore, we can derive an overall rank defined as:


(10) $$\begin{array}{l} {𝔎}_{b}=rank\left(b:x_{b2},\;\ldots ,\;x_{bK}\;\right) \end{array}$$

Once *r*_*b*1_ and 𝔎_*b*_ are calculated, they can be replaced into (9):


(11) $$\begin{array}{l} K\left({𝔎}_{b},r_{b1}:d\right)=\sup\left\{K>0|({𝔎}_{b}+Kd_{b},r_{b1}+Kd_{b1})\in T\right\} \end{array}$$

We can also set signs of our directionalcan also set signs of our directional vector by vector by choosing to sort ranks in ascending or descending orders. This is done by transforming individual distribution of variables into a single uniform distribution regardless of asymmetries in the original distributions. Therefore, this model significantly reduces dimensionality of datasets and increases computational capacity compared to the mathematical programming models or the nonparametric maximum likelihood models typically used in the literature (see for instance, Banker and Morey, 1986; Seiford and Thrall, 1990; and Kumbhakar et al., 2007). The directional vector in our case is *d* = (*d*_*b*_, *d*_*b*1_) = (−1, −γ_*b*_). There are negative signs because both ranks are sorted in an ascending order, i.e., 1 is allocated to the most preferred entity and so on for the rest of the decision-making units. The magnitude of the direction *d*_*b*1_, is $\gamma_{b}=\frac{\left({𝔎}_{b}-{𝔎}_{eff}\right)}{\left(r_{b1}-r_{eff,1}\right)}$ that shows the level of association between the individual rank and the overall rank for the entity *b*, and scales the overall rank proportionally to the individual rank. The ranks for an entity that is located on the frontier curve is given by *r*_*eff*, 1_ and 𝔎_*eff*_.

According to Fernandez et al. (2000), the frontier is not known exactly and estimation usually leads to the two-sided error terms (see also Layer et al., 2020). On the other hand, Land et al. (1993) note that the most productive decision-making units close to the frontier are assumed to represent frontier decision-making units. Instead, in γ_*b*_ we assume an ideal condition where there is a hypothetical decision-making unit that is located on the frontier. In this case the individual rank for this entity is *r*_*eff*, 1_ = 1 and the overall rank is 𝔎_*eff*_ = 1.

In the literature, the distance β(*x*_*b*_, *y*_*b*_:*d*_*x*_) is calculated using linear programming or maximum likelihood estimation methods. To obtain the ranked equivalent to β(*x*_*b*_, *y*_*b*_:*d*_*x*_, *d*_*y*_), i.e., the ranked distance *K*(𝔎_*b*_, *r*_*b*1_:*d*) in equation 11, we use the following


(12) $$\begin{array}{l} K\left({𝔎}_{b},r_{b1}:d\right)=\frac{\left({𝔎}_{b}-{𝔎}_{eff}\right)}{\gamma_{b}}=\left(r_{b1}-r_{eff,1}\right) \end{array}$$

Subject to


(13) $$\begin{array}{l} \underset{{𝔎}_{b},r_{b1}}{argmax}\left(\sqrt{{\left({𝔎}_{b}-{𝔎}_{eff}\right)}^{2}+{\left(r_{b1}-r_{eff,1}\right)}^{2}}\right),\;b=1,\ldots ,c,...,B \end{array}$$

K(𝔎_*b*_, *r*_*b*1_:*d*) is a locus i.e., if γ_*b*_ = 1, the difference between the best rank and the worst rank is the same for both ranks (single rank and overall rank). If γ_*b*_ is larger than 1, then reduction of the gap between worst rank and better rank in innovation (in other words spend more in intangibles) implies an even larger reduction in the gap between worst and better ranks in the overall rank. If γ_*b*_ is smaller than 1, then we are in the opposite situation.

## Empirical analysis: Data, operationalization, and methodology

### Data

We source the data from Orbis BankFocus (formerly Bankscope)^1^. We include all available financial institutions as long as they are parent or holding companies because the majority of investment in innovation^2^ is usually performed by parent companies. For example, Goldman Sachs Group recorded $4 bn of intangible assets while its daughter bank Goldman Sachs Bank recorded only $65 mln, as reported by BankFocus for 2019. Moreover, from an accounting point of view, bank accounts are typically consolidated within the holding company's consolidated financial statements. In the case of unconsolidated reports, the proportion of intangible assets of the subsidiary company in the parent's holdings usually is negligible.

To identify the parent company, we have selected financial institutions whose ultimate owners can be identified within the database. Additionally, as in Kenjegalieva et al. (2009) and Tsionas et al. (2015), we carefully scrutinized accounting standards to avoid duplicate data for the same bank. Orbis database reports two different accounting standards: GAAP and IFRS. We use US GAAP accounting standard for American banks and IFRS accounting standard for European banks.

The full sample consists of a dataset covering the period from 2010 to 2019. Our data consist of four input variables: total assets, number of employees, other intangible assets (proxy for innovation) and mortgage servicing rights (MSRs); and two output variables: gross loans and NPL ratio (bad output). A list of these variables with their definitions are provided in Table 1 and descriptive statistics in Table 2A. We normalize input variables with gross loans and then standardized them with the sample mean of each input. In total we have data for 1,887 US banks and 388 EU banks. Data for MSRs available only for 64 US banks. Hence, we assume MSRs are equal to zero for the rest of the US financial intermediaries. Since the US financial intermediaries capitalize MSRs with other intangible assets, we subtracted MSRs from other intangible assets.^3^

**Table 1:** List of variables and their definitions.

**Variable**	**Label**	**Definition**	**Orbis Line #**
**Outputs**			
Loans	Gross loans and advances to customers	Gross amount of Mortgage loans, Consumer loans, Corporate loans and Other loans	51,350
NPL	Impaired loans/Gross customer loans & advances	Impaired loans as a percent of Gross customer loans and advances computed based on impaired loans methodology for the respective country.	99,280
**Inputs**			
Total assets	Total assets	Sum of on balance sheet asset	52,600
Employment	Number of employees	Total number of employees or FTE reported at the end of reporting period	80,000
Innovation	Other Intangible assets (proxy for innovation)	All other intangibles other than Goodwill, on net basis	52,400
MSR	Mortgage servicing rights, balance at end of period	Mortgage Servicing Rights represent net income to be received for servicing an existing portfolio of mortgage loans owned by others (Kohlbeck, 2004).	Bankfocus/Moody's Investor Service

Table 2Data analysis: descriptive statistics and independent samples test between US and EU banks.

**Table:** 

	**Country**	**Number of observations:**			**Mean**	**Std. deviation**	**Std. error mean**
		**Total available**	**Specification with**		**Total available**		
			**Intangible assets**	**Non-performing loans (NPL)**	
**(A) Descriptive statistics for banks of two countries: US and EU**							
1	2	3	4	5	6	7	8
Number of Employees	USA	8,589	5,513	8,162	5.499	1.777	0.019
	EU	2,669	2,520	2,322	7.375	1.829	0.035
Total Assets	USA	8,589	5,513	8,162	14.024	1.926	0.021
	EU	2,669	2,520	2,322	16.199	2.120	0.041
Other Intangible Assets	USA	5,709	5,513	-	8.129	2.588	0.034
	EU	2,754	2,520	-	9.520	2.800	0.053
NPL	USA	8,337	-	8,162	2.055	3.666	0.040
	EU	2,426	-	2,322	9.354	12.429	0.252
Loans	USA	8,668	5,513	8,162	13.484	1.920	0.021
	EU	2,796	2,520	2,322	15.466	2.428	0.046
MSR^*^	US	650			3.022	2.475	0.097

**Table:** 

		**Levene's Test for**		***t*** **-test of Equality of Means**						
		**Equality of Variances**								
									**95% confidence interval**	
									**of the difference**	
		**F-stat**	**Sig**.	**t-stat**	**df**	**Sig. (2-tailed)**	**Mean Difference**	**Std. Error Difference**	**Lower**	**Upper**
**1**	**2**	**3**	**4**	**5**	**6**	**7**	**8**	**9**	**10**	**11**
**(B) Independent samples test between US and EU banks**										
Employees	Equal variances assumed	6.991	0.008	−47.284	11,256	0.000	−1.875	0.039	−1.952	−1.797
	Equal variances not assumed			−46.567	4345.782	0.000	−1.875	0.040	−1.954	−1.796
Total assets	Equal variances assumed	36.054	0.000	−49.737	11,256	0.000	−2.175	0.043	−2.26	−2.089
	Equal variances not assumed			−47.285	4126.536	0.000	−2.175	0.046	−2.265	−2.085
Other intangible assets	Equal variances assumed	40.943	0.000	−22.559	8461	0.000	−1.392	0.062	−1.512	−1.271
	Equal variances not assumed			−21.948	5074.056	0.000	−1.392	0.063	−1.516	−1.267
NPL	Equal variances assumed	2467.089	0.000	−47.051	10761	0.000	−7.298	0.155	−7.602	−6.994
	Equal variances not assumed			−28.565	2548.848	0.000	−7.298	0.255	−7.799	−6.797
Loans	Equal variances assumed	185.306	0.000	−44.315	11,462	0.000	−1.981	0.045	−2.068	−1.893
	Equal variances not assumed			−39.352	3983.357	0.000	−1.981	0.050	−2.080	−1.882

All data are in natural logs, except for non-performing loans (NPL) which is a ratio of impaired loans to gross customer loans. The data spans from 2010 to the most recent available: 2019 (10 years in total). Missing observations are automatically excluded from the individual variables. Total assets exclude intangible assets.

* Since MSRs do not represent innovation but capitalized with other intangible assets by the US financial intermediaries, it was suggested that MSRs should be subtracted from other intangible assets. We would like to thank an anonymous reviewer for pointing to this issue. There are 64 US banks with MSR, for the rest of the banks we assume MSRs equal zero.

We consider three specifications: (1) innovation (proxied by intangible assets), (2) non-performing loans (a ratio of impaired loans to gross customer loans and advances) and (3) a sample drawn from a Gaussian distribution. For the former two specifications, we omit a bank-year observation where there was a missing observation within a single variable. As a result, the final sample sizes for these two specifications are smaller than the full sample and differ between each other and vary from year to year.

### Methodology operationalisation

The methodology applied to the specifications are operationalised in the following way:

Step 1: Calculate an individual rank (*r*_*b*1_) based on the investment in intangible assets in Model 1. We do the same for the non-performing loans in Model 2 and for one of the random variables in Model 3.
Step 2: Use the Promethee outranking method to generate the overall rank. We use the number of employees and total assets as the input variables.
Step 3: This is the final step when we use (12) and (13) to get *K*(𝔎_*b*_, *r*_*b*1_:*d*) and γ_*b*_.

### Data analysis: Independent samples test and levene's test

Before proceeding to estimation of the two models, we examine the data for the US and EU banks. We expect that due to globalization, financial markets in both regions converge. Therefore, we compare the first moments of each variable between two regions. We use an independent samples test to check whether the means of the variables for the US and EU banks are statistically different from each other (and Welch, 1947; see Imbens and Kolesár, 2016). This test assumes equal variance of the variables across the two samples; we test whether the variances are indeed equal by using the Levene's test (Levene, 1960; Brown and Forsythe, 1974).

The results of the independent samples test are presented in Table 2B. This table shows that the means of the variables in the US and EU are statistically different from each other. The results are consistent under both assumptions on variances. For example, the t-statistics for “*Employees”* computed with assumption of the equal variances is −47.284 (see Table 2B, column 5). At the same time, t-statistics computed under the assumption of unequal variances is −46.567. In both cases, they are significant at 1% level. Additionally, column 8 in Table 2B indicates that the mean difference for “*Employees”* is −1.875 and std. error differences are 0.039 and 0.040, respectively. Comparable results are obtained for “*Total Assets,” “Other Intangible Assets,”* and “*Non-Performing Loans.”* The Levene's test also shows that the variances of the variables across the two data-sets are statistically different.

Observation of the mean differences (Table 2B, Column 8) indicates that all variables, at the sample mean, are larger for the EU banks compared to the US banks. One interesting point to note is that the magnitude of the mean difference for “*Non-Performing Loans”* is much larger than for the rest of the variables. This result possibly suggests that the US banks are more competitive than EU banks and they maximize their efficiency by utilizing technologies (such as ICT) that reduce other costs including non-performing loans.

## Estimation results

### Model 1: Innovation expenditure and combined rank

Table 3 reports the results of Model 1 that focuses on innovation expenditure. One of the key parameters to focus on, along with the distance K(𝔎_*b*_, *r*_*b*1_:*d*), is γ that shows the magnitude of the change in the overall rank in response to the unit change of the innovation rank. Since we ranked innovation in ascending order, underperforming units are at the bottom of the sample (in terms of ranks). However, inspection of K(𝔎_*b*_, *r*_*b*1_:*d*) in Table 3 indicates that inefficient units do not have the worst individual or overall ranks (see respective panels A and B, Table 2). A combination of the ranks suggests the units are inefficient. For example, for the US banks K(𝔎_*b*_, *r*_*b*1_:*d*) = 416 and γ = 0.947 in 2019, and this outcome translates into the individual rank of 417 and the overall rank of 394 compared to the maximum possible rank of 422 in that year. Similar results are observed for the rest of the banks and years in the US as well as the EU.

**Table 3:** K(𝔎_*b*_, *r*_*b*1_:*d*) and gamma in the model with innovation for US and EU banks.

			**2019**	**2018**	**2017**	**2016**	**2015**	**2014**	**2013**	**2012**	**2011**	**2010**
**1**	**2**	**3**	**4**	**5**	**6**	**7**	**8**	**9**	**10**	**11**	**12**	**13**
US	**(A) Ranked distance and gamma**											
	Inefficient unit	K(𝔎_*b*_, *r*_*b*1_:*d*)	416	394	559	518	524	658	656	619	537	500
		Gamma, γ	0.947	1.036	0.986	1.028	0.927	0.972	1.000	1.003	1.011	1.038
	Most efficient unit	K(𝔎_*b*_, *r*_*b*1_:*d*)	12	7	6	14	12	11	36	18	7	8
		Gamma, γ	0.091	0.429	0.200	0.077	0.250	2.091	0.278	0.222	0.167	0.143
	**Number of Units**		422	419	564	541	527	670	658	623	545	521
EU	**(B) Ranked distance and gamma**											
	Inefficient unit	K(𝔎_*b*_, *r*_*b*1_:*d*)	226	273	249	255	278	272	274	205	200	61
		Gamma, γ	0.960	1.059	1.225	1.180	1.054	1.088	0.880	0.944	0.965	0.943
	Most efficient unit	(𝔎_*b*_, *r*_*b*1_:*d*)	32	13	1	1	1	1	1	7	34	8
		Gamma, γ	0.609	3.346	1.000	2.000	2.000	18.500	14.000	4.286	0.412	0.143
	**Number of Units**		241	306	306	303	296	300	285	214	206	63

While K(𝔎_*b*_, *r*_*b*1_:*d*) of inefficient units depend on the number of banks during analyzed period, we expect K(𝔎_*b*_, *r*_*b*1_:*d*) to be close to unity for the most efficient units. Table 3 indicates that this is not always the case. For instance, the efficient bank's K(𝔎_*b*_, *r*_*b*1_:*d*) in the US is as worse as 14; nonetheless the value of γ is 0.077 (in 2016) and converted into ranks they give 15 and 1 (out of 541) for the individual and overall ranks, respectively. At the same time, the efficient EU banks' worst K(𝔎_*b*_, *r*_*b*1_:*d*) is observed in 2011 with K(𝔎_*b*_, *r*_*b*1_:*d*) = 34 and γ = 0.412. In general, K(𝔎_*b*_, *r*_*b*1_:*d*) for the efficient unit is lowest in the US than in the EU (particularly so at the beginning of the period). At the same time, for the EU K(𝔎_*b*_, *r*_*b*1_:*d*) = 1 can be observed for several periods. We conjecture that this result is due to the strong competition preventing banks in the top tier from having the highest ranks.

From panels A and B of Table 3, it can be observed that γs for inefficient units both in the US and the EU fluctuates around 1. Moreover, in 2013, γ equals one in the US. This indicates that in order to reach the frontier point the bank needs to have equi-proportional changes in both ranks. The maximum absolute value for γ in the US is observed in 2010 (γ = 1.038). For the EU banks the variation in γ is slightly higher than for the US banks but broadly similar. Among the most efficient units, we find a large variation in γ. For example, the lowest absolute value of γ is equal to 0.077 for the efficient US bank in 2016 and for the EU banks it is 0.143 in 2010. The largest γ across two economies is in the EU with γ = 18.500 in 2014. For the US banks the largest γ is observed in 2014 with γ = 2.091.

**Figure 1:**
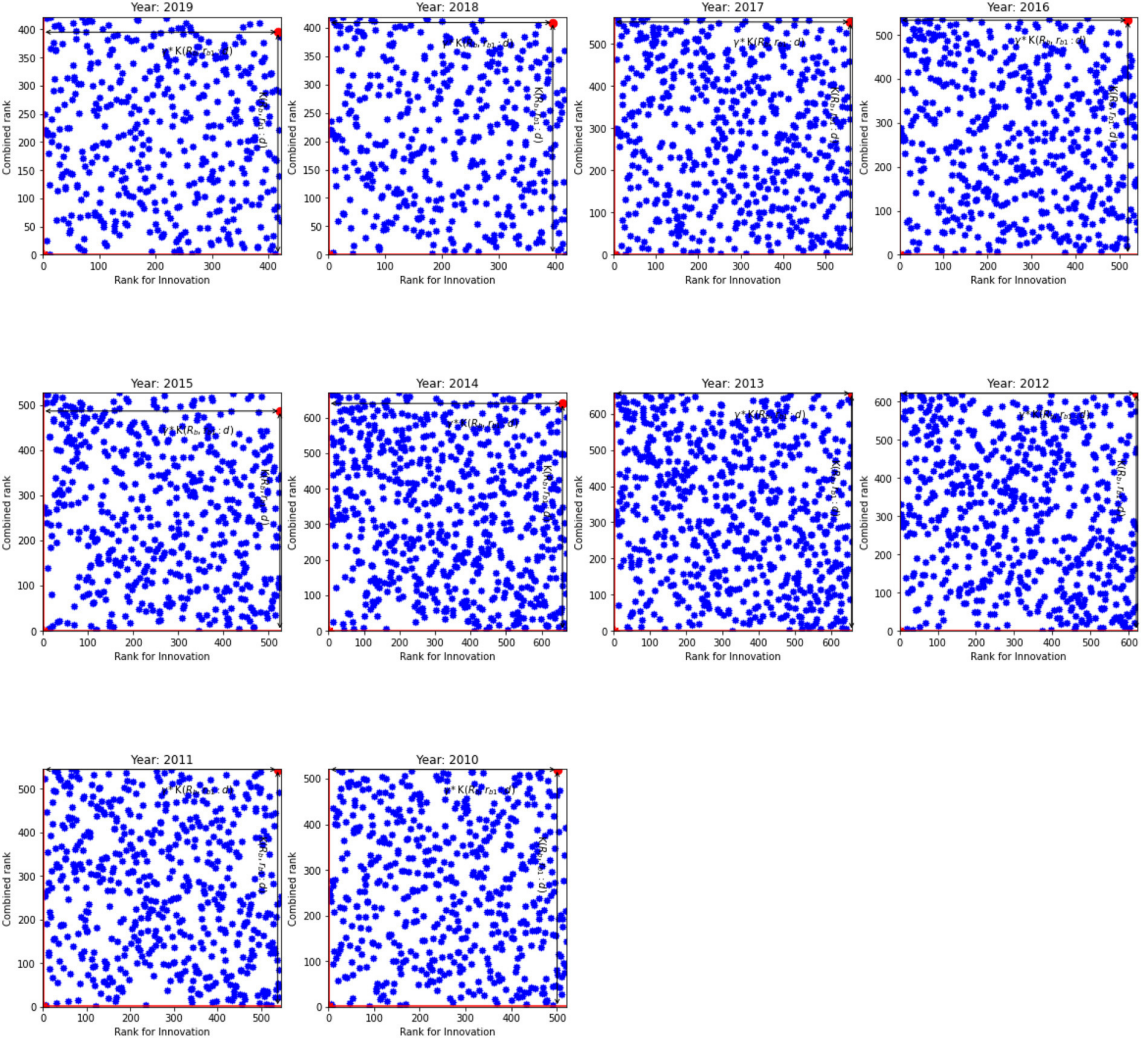
The innovation rank against combined rank for the US. K(𝔎_*b*_, *r*_*b*1_:*d*) and γ*K(𝔎_*b*_, *r*_*b*1_:*d*) for the inefficient unit.

**Figure 2:**
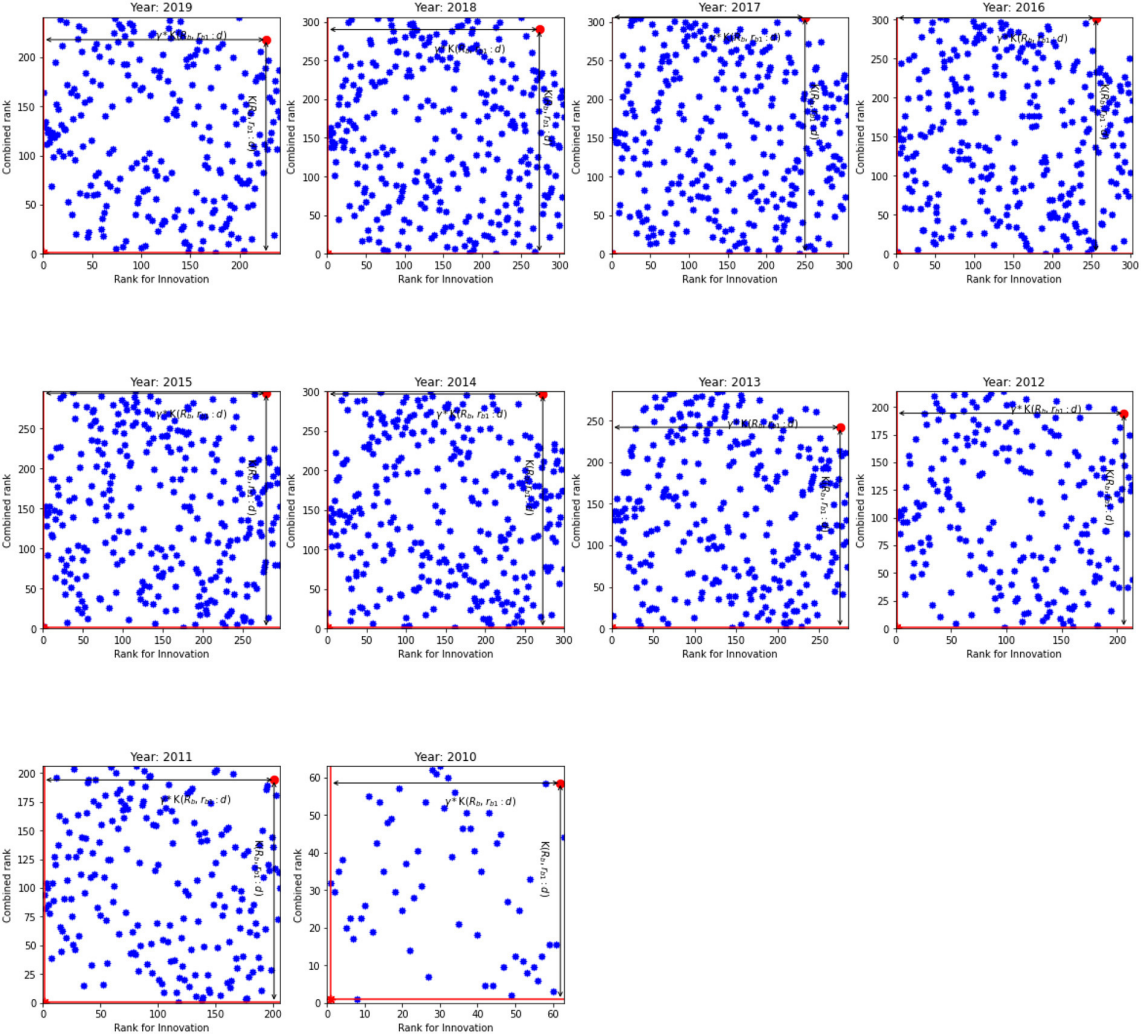
The innovation rank against combined rank for the EU. K(𝔎_*b*_, *r*_*b*1_:*d*) and γ*K(𝔎_*b*_, *r*_*b*1_:*d*) for the inefficient unit.

In spite of (𝔎_*b*_, *r*_*b*1_:*d*) and γ show the fastest path to *r*_*eff*, 1_ = 1 and K_*eff*_ = 1, Figures 1, 2 indicate that distribution of ranks are uniform and independent of each other. Given the same overall rank, ranks for innovation are different across banks. Our results indicate that there are four types of banks in our sample. First, the banks where the innovation rank is positively correlated with the overall rank, i.e., the high overall rank is correlated with high innovation rank. These banks are located in the SW quadrant. The second type of banks exhibits a negative correlation between two ranks: their overall ranks are low while still exhibiting high innovation ranks. They can be observed in the WN quadrant. The third type of banks have high overall rank but low innovation rank. Finally, the fourth group in NE quadrant have the worst ranks both for the innovation rank and the overall rank. The least efficient banks belong to this group. For example, banks located in South-West (SW) and North-East (NE) quadrants show that their innovation ranks are in line with their overall ranks. These are the best and the worst banks, respectively. However, an interesting picture can be observed from West-North (WN) and East-South (ES) quadrants. Banks located in WN quadrant have high innovation ranks, that is comparable with banks from SW quadrant, nonetheless they have low overall ranks.

Moreover, we find that the inefficient units do not have the worst individual or combined ranks. Rather a combination of them scores the units as inefficient. Similar results are observed for all years in the US as well as the EU. At the same time, we expect K(𝔎_*b*_, *r*_*b*1_:*d*) of the efficient units to be close to unity. However, our results indicate that this is not the case. In general, the K(𝔎_*b*_, *r*_*b*1_:*d*) for the efficient units are worse in the US than in the EU.

### Model 2: Non-performing loans and combined ranks

In case of non-performing loans (NPL), K(𝔎_*b*_, *r*_*b*1_:*d*) for inefficient units behaves similar as in spec 1 (see Table 4). In other words, the worst ranked banks do not have the lowest K(𝔎_*b*_, *r*_*b*1_:*d*). However, γ for the inefficient unit in NPLs is closer to unity than γ for the inefficient unit in innovation. Moreover, there is less variation in γ. This result indicates that, in general, a change in one of the ranks is accompanied by a proportional change in the other rank.

**Table 4:** K(𝔎_*b*_, *r*_*b*1_:*d*) and gamma in the model with non-performing loans for US and EU banks.

			**2019**	**2018**	**2017**	**2016**	**2015**	**2014**	**2013**	**2012**	**2011**	**2010**
1	2	3	4	5	6	7	8	9	10	11	12	13
US	**(A) Ranked distance and gamma**											
	Inefficient unit	K(𝔎_*b*_, *r*_*b*1_:*d*)	574	579	779	745	742	1,020	981	961	839	811
		Gamma, γ	1.000	1.003	0.995	1.012	1.011	1.017	1.036	1.015	0.970	0.993
	Most efficient unit	K(𝔎_*b*_, *r*_*b*1_:*d*)	8.5	12	20	14.5	19	2	9	24.5	14	35.5
		Gamma, γ	1.176	1.333	1.000	0.897	1.000	5.500	0.333	0.571	2.071	0.225
	**Number of Units**		576	582	781	755	751	1,038	1,017	984	857	821
EU	**(B) Ranked distance and gamma**											
	Inefficient unit	K(𝔎_*b*_, *r*_*b*1_:*d*)	228	289	278	273	276	271	258	192	179	51
		Gamma, γ	1.009	1.003	1.004	0.956	0.996	0.976	1.000	1.000	1.006	1.039
	Most efficient unit	K(𝔎_*b*_, *r*_*b*1_:*d*)	3	5	7	7	8	11	10	5	4	1
		Gamma, γ	2.333	0.800	0.167	1.000	1.000	0.318	0.050	0.250	0.250	2.500
	**Number of Units**		231	291	280	281	277	274	260	193	181	54

Table 4 also shows the results for efficient units. Distances in the most efficient units for non-performing loans are much closer to the frontier point. For example, while K(𝔎_*b*_, *r*_*b*1_:*d*) for innovation was relatively far from 1 [the closest to one is K(𝔎_*b*_, *r*_*b*1_:*d*) = 7], for NPL this value equals to 0.5. At the same time, there is a variation in the behavior of γ but it is mostly <1. Exception happened when γ was equal to 12 and this indicates that there was a large difference between the two ranks in 2010.

**Figure 3:**
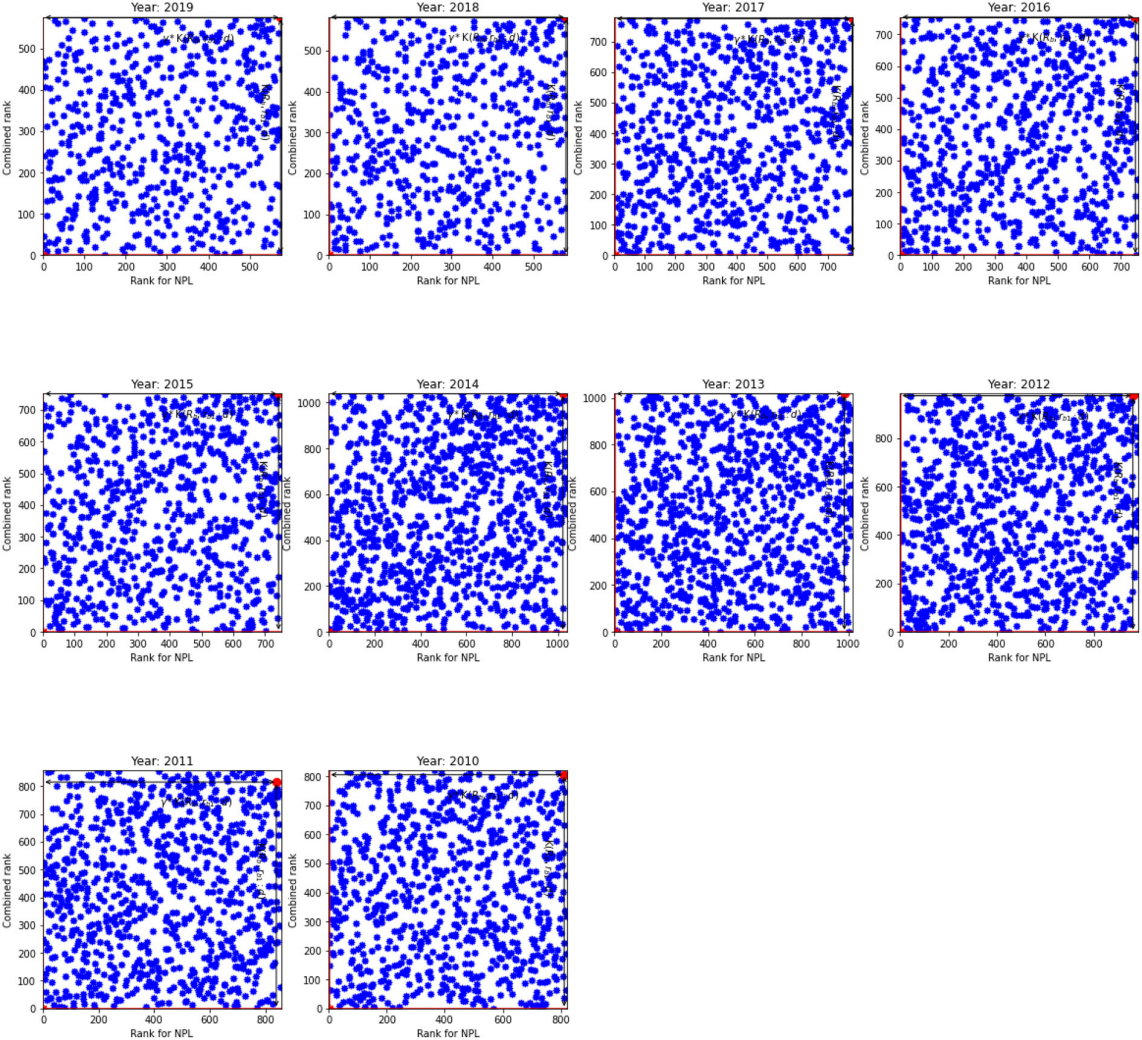
The NPL rank against combined rank for the US. K(𝔎_*b*_, *r*_*b*1_:*d*) and γ*K(𝔎_*b*_, *r*_*b*1_:*d*) for the inefficient unit.

**Figure 4:**
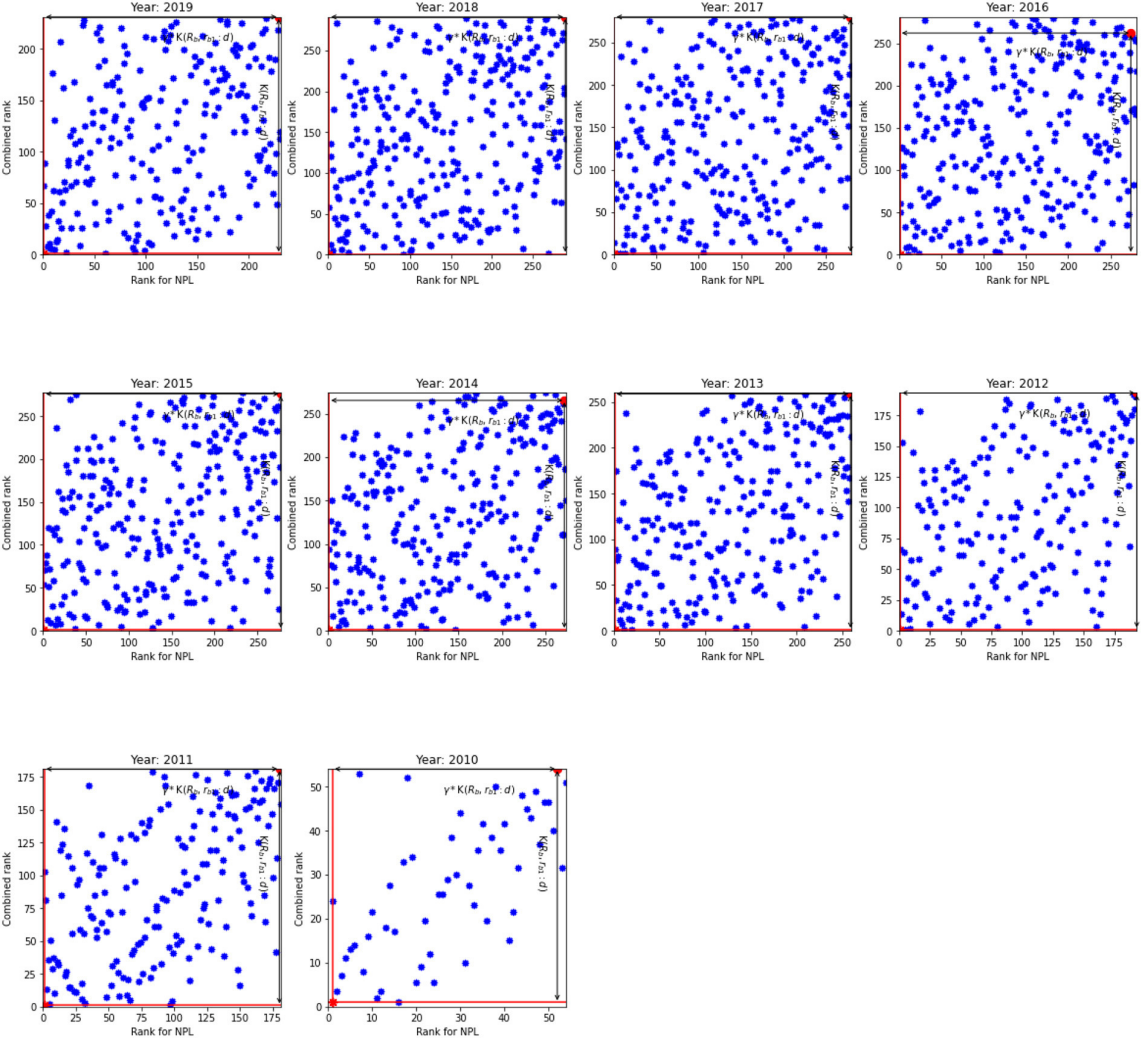
The NPL rank against combined rank for the EU. K(𝔎_*b*_, *r*_*b*1_:*d*) and γ*K(𝔎_*b*_, *r*_*b*1_:*d*) for the inefficient unit.

The results for non-performing loans given in Table 4 broadly resemble the results in Table 3. However, a visual inspection of Figures 3, 4 show that the relationship between the NPL rank and the overall rank differs from the relationship between the innovation rank and the overall rank observed in Figures 1, 2. Indeed, there is a positive linear relationship between the NPL rank and the combined rank: as the NPL rank improves so does the combined rank. This relationship can be seen in Figure 3 that shows the NPL rank and the combined rank for the US. It shows that most banks are located along the primary diagonal line. The most inefficient unit is in NE quadrant and the most efficient bank at the bottom of the diagonal line (SW quadrant).

Overall, the number of analyzed banks in the EU is much lower than the number of banks in the US: by approximately four to five times. As a result, we observe relatively low number of banks in the upper and lower diagonals (WN and ES quadrants) and hence, the diagonal line is more prominent for the EU than for the US banks. Based on the results for non-performing loans, we argue that the banks that are able to screen out risky borrowers efficiently—therefore, they have higher NPL ranks—are able to reduce costs without affecting the quality of loans and hence they are more competitive that leads to improvement in the overall rank. In addition, smaller NPL ratio means that banks need less provision for future loan losses that increases their loan base; and leads to lower interest rates and whence they can provide cheaper loans.

### Model 3: Ranks based on random samples

**Figure 5:**
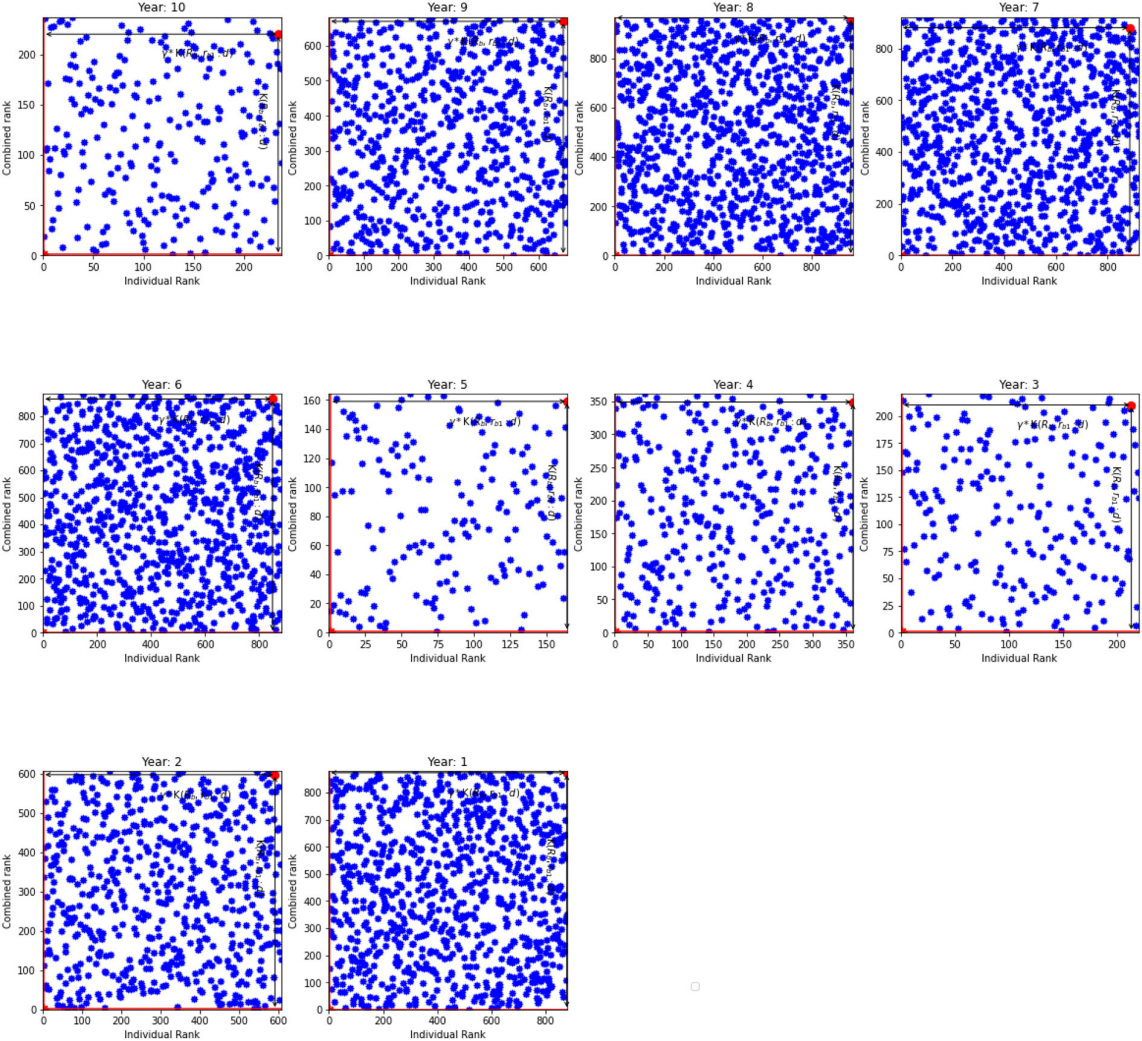
The individual rank against combined rank (from random normal samples). K(𝔎_*b*_, *r*_*b*1_:*d*) and γ*K(𝔎_*b*_, *r*_*b*1_:*d*) for the inefficient unit.

In this section we provide results of the model with randomly generated variables. Each variable has mean zero but differs in the variance. We split the simulated data into ten distinct periods to emulate the actual data. Table 5 shows outcome of this model. According to the table the inefficient units do not have the worst ranks. This is similar to previous specifications, whereas a combination of overall and individual ranks marks the units as inefficient. For instance, K(𝔎_*b*_, *r*_*b*1_:*d*) = 233 and γ=0.940 for the period 10 that suggests that the overall rank is 219 and individual rank is 234. Dynamics of the parameter γ, according to Table 5, show that γ gravitates around unity and indicates a proportional change in ranks. Additionally, Table 5 shows the results for the efficient units. Distances K(𝔎_*b*_, *r*_*b*1_:*d*) in the most efficient units varies from 1 to 24 with γs as low as 0.27 and as high as 18. Since we are using random sample, Figure 5 shows that the units are scattered randomly across the graphs. Overall, the results are similar to results from models 1 and 2.

**Table 5:** K(𝔎_*b*_, *r*_*b*1_:*d*) and gamma in the model with random samples from a normal distribution.

			**10**	**9**	**8**	**7**	**6**	**5**	**4**	**3**	**2**	**1**
1	2	3	4	5	6	7	8	9	10	11	12	13
**Random normal sample**	**Ranked distance and gamma**											
	Inefficient unit	K(𝔎_*b*_, *r*_*b*1_:*d*)	233	670	957	885	849	163	360	212	590	877
		Gamma, γ	0.940	0.999	1.006	0.993	1.016	0.969	0.967	0.986	1.012	0.994
	Most Efficient Unit	K(𝔎_*b*_, *r*_*b*1_:*d*)	1	11	36	24	12	1	20	18	37	20
		Gamma, γ	18.000	1.773	0.556	0.708	2.833	14.000	0.550	0.667	0.270	1.400
	**Number of Units**		237	682	967	921	882	164	361	220	607	879

## Conclusions

In this paper, we have proposed a new methodology that combines standard production theory with MCDA methods to rank banks based on their capability of using investment in digital technologies to reduce the other inputs' usage, for a given level of output. To capture the fact that contractions of inputs may be associated to increases of the remaining input, we propose to replace the inputs with ranks which capture the fact that the inputs move in different directions.

To exemplify the methodology, we have applied it to a sample of US and EU banks sourced from Orbis BankFocus for 2010 to 2019. We have considered three models. The first one focuses on investment on intangible assets as a proxy of innovation (proxied by intangible assets). The second one focuses on non-performing loans (a ratio of impaired loans to gross customer loans and advances) and allows to illustrate how our approach can be used for other types of variables while the third model focuses on a sample drawn from a Gaussian distribution. Results suggest that banks can be sorted in four groups according to their capability of using investment in new technologies to reduce other inputs' usage, for a given level of output.

Our methodology can help banks to quantify the impact of their investment in digital technologies and can take actions that improve its impact. Conversely, financial investors and other stakeholders can use it to detect how efficiently the bank can utilize its digital technologies. And, since these activities have direct implications on future financial standing of the bank, the results can help forecast banks' future financial performance.

## Data availability statement

The original contributions presented in the study are included in the article/supplementary materials, further inquiries can be directed to the corresponding authors.

## Author contributions

All authors listed have made a substantial, direct, and intellectual contribution to the work and approved it for publication.

## Conflict of interest

The authors declare that the research was conducted in the absence of any commercial or financial relationships that could be construed as a potential conflict of interest.

## Publisher's note

All claims expressed in this article are solely those of the authors and do not necessarily represent those of their affiliated organizations, or those of the publisher, the editors and the reviewers. Any product that may be evaluated in this article, or claim that may be made by its manufacturer, is not guaranteed or endorsed by the publisher.

## Footnotes

^1^This database is used, for example, by Fiordelisi et al. (2011) and Tsionas et al. (2015) to obtain data for EU-15 and EU-26 countries, respectively.

^2^See for example accounting rules on other intangible assets in IFRS – IAS 38, FASB ASC – Topic 350 and FRS102 – Section 18, but cf. SFFAS 10.

^3^We would like to thank an anonymous reviewer for pointing to this issue.
